# Technological innovation in healthcare: challenges, opportunities and impact on sustainability

**DOI:** 10.3389/fdgth.2026.1720615

**Published:** 2026-07-08

**Authors:** María Vallet-Regí, Manuel Doblaré, José A. Garrido, Almudena Fuster-Matanzo, Ángel Alberich-Bayarri, María Elena Hernando, Cecilia E. García Cena, Marie Destarac Eguizabal, Emilio Bouza

**Affiliations:** 1 Universidad Complutense de Madrid, Madrid, Spain; 2 Instituto de Investigación Sanitaria Hospital 12 de Octubre, Madrid, Spain; 3 Fundación Ramón Areces, Madrid, Spain; 4 Centro de Investigación Biomédica en Red en Bioingeniería, Biomateriales y Nanomedicina (CIBER-BBN), Instituto de Salud Carlos III, Madrid, Spain; 5 Instituto Universitario de Investigación en Ingeniería de Aragón (I3A), Universidad de Zaragoza, Zaragoza, Spain; 6 Instituto de Investigación Sanitaria Aragón (IISA), Zaragoza, Spain; 7 ICREA, Barcelona, Spain; 8 Catalan Institute of Nanoscience and Nanotechnology (ICN2), CSIC and The Barcelona Institute of Science and Technology, Barcelona, Spain; 9 Quibim S.L, Valencia, Spain; 10 Centro de Tecnología Biomédica, Universidad Politécnica de Madrid, Madrid, Spain; 11 Universidad Politécnica de Madrid, Escuela Técnica Superior de Ingeniería y Diseño Industrial, Madrid, Spain; 12 Centro de Automática y Robótica (ETSIDI-UPM-CSIC), Madrid, Spain; 13 OWSD Guatemala, Guatemala, Guatemala; 14 ABB Spain, Madrid, Spain

**Keywords:** healthcare, innovation, policies, sustainability, technology

## Abstract

Healthcare technologies are increasingly reshaping how diseases are detected, monitored, treated, and managed across healthcare systems. Advances in artificial intelligence (AI), digital health, remote monitoring, advanced medical devices, and data-driven clinical infrastructures are creating important opportunities to improve prevention, diagnostic accuracy, personalisation of care, workflow efficiency, and long-term healthcare sustainability within the framework of predictive, preventive, personalized, and participatory medicine (4P Medicine). However, despite growing technological sophistication and investment, many innovations fail to achieve scalable and sustainable implementation in real-world clinical environments. This narrative review critically examines the systemic factors that condition the successful translation of healthcare technologies into routine clinical practice, with particular emphasis on the European and Spanish contexts. Rather than focusing exclusively on technological performance, the review analyses the broader regulatory, organisational, financial, ethical, and governance challenges that shape implementation. Key areas discussed include technology transfer, regulatory frameworks, health data governance, and the organisational challenges associated with implementing AI-driven healthcare technologies. The central argument of this review is that the real-world impact of healthcare innovation depends less on technological capability itself than on the capacity of healthcare systems to support validation, regulation, implementation, workforce adaptation, interoperability, and long-term governance. Consequently, the principal challenge for contemporary healthcare systems is no longer simply how to develop new technologies, but how to integrate them safely, equitably, and sustainably into routine clinical practice.

## 1 Introduction

Technological innovation has historically played an important role in progress across healthcare systems, influencing disease prevention, diagnosis, treatment, and healthcare system organization. Its influence on healthcare has been particularly significant, extending beyond incremental improvements to influence medical practice, patient management, and the capacity of healthcare systems to respond to emerging global health challenges. This interplay between technology and healthcare increasingly shapes contemporary medical practice, directly influences patient wellbeing and impacts the organization and sustainability of healthcare systems.

The exponential growth of technologies applied to healthcare is reflected in the investment and widespread adoption of innovative solutions across hospitals, primary care centres and even in patients' homes. This expansion has been driven by decades of technological advances across multiple fields. In particular, the refinement of advanced medical imaging techniques, such as magnetic resonance imaging (MRI), computed tomography (CT) and positron emission tomography (PET), together with breakthroughs in genomics and the power of medical informatics, has enabled earlier and more precise diagnosis as well as more personalized therapeutic strategies. A clear indicator of this expansion is the global digital health market, which was valued at $197 billion in 2025, and is projected to reach $258.25 billion by 2029, reflecting a robust compound annual growth rate of 6.88% (1). Likewise, investment in research and development in this field is growing steadily. Besides pharmaceutical improvements, emerging technologies, such as telemedicine, artificial intelligence (AI), new medical imaging techniques, organ preservation, enhanced sensors and devices, new biomaterials and implants, tissue engineering systems, controlled drug delivery mechanisms, and digitized health management platforms, are substantially transforming the landscape of modern medicine.

However, despite the growing technical sophistication and investment associated with healthcare technologies, their successful translation into scalable and sustainable real-world healthcare impact remains highly variable. Beyond technological performance, implementation and large-scale integration depend on broader systemic factors including regulatory frameworks, clinical validation pathways, interoperability, workforce adaptation, reimbursement structures, technology transfer capacity, and patient and clinician adoption. In many cases, technologies with promising technical capabilities fail to progress beyond pilot phases or encounter substantial barriers to widespread implementation. These challenges are particularly relevant in healthcare systems facing increasing pressure to balance innovation, patient safety, equitable access, and long-term sustainability.

Within this context, the European setting represents a particularly relevant environment for examining the opportunities and limitations associated with healthcare innovation, given the increasing complexity of regulatory requirements, healthcare system sustainability challenges, and technology transfer processes. More specifically, Spain constitutes an illustrative case regarding barriers and opportunities related to public-private collaboration, innovation funding, technology transfer, and adoption within public healthcare systems.

This review examines a selection of the principal technological advancements—outside chemical and biological treatments—that are substantively changing the healthcare sector. It critically analyses their development lifecycle, from conceptualization through regulatory certification to commercialisation and examines the systemic factors that condition real-world impact. A central focus is the relationship between technological innovation and the long-term sustainability of healthcare systems, with particular reference to the European context, and, more specifically, to Spain. Ethical and regulatory challenges in data governance and AI deployment, alongside the growing paradigm of patient empowerment facilitated by connected devices, are examined in this light.

The central argument of this review is that the real-world impact of healthcare technologies depends not primarily on their technical performance but on a set of systemic factors: the adequacy of validation and regulatory frameworks, the maturity of technology transfer mechanisms, the availability of sustained funding, the training of clinical professionals, and the governance of health data. Addressing the question of why promising technologies frequently fail to achieve scalable, sustainable clinical impact requires examining these structural conditions rather than the technologies in isolation. This paper aims to offer an analytically grounded perspective that is useful to decision-makers, policymakers, and researchers working at the intersection of innovation and healthcare systems sustainability in the European and Spanish context.

## 2 Methodological approach

This article was developed as a narrative review combined with an expert policy and implementation perspective on the integration of emerging technologies into healthcare systems. Rather than following a formal systematic review methodology, the manuscript aims to provide a thematic and critical synthesis of key technological, regulatory, organisational, ethical, and translational challenges shaping the implementation of AI-driven and digital health technologies. The literature discussed throughout the manuscript was selected based on relevance to the conceptual objectives of the review, with particular emphasis on healthcare sustainability, implementation barriers, governance challenges, innovation ecosystems, and the European and Spanish healthcare contexts. The objective of the review is therefore not to provide an exhaustive evaluation of all available evidence, but rather to develop an integrative and critical perspective on the systemic factors influencing the translation of technological innovation into sustainable healthcare impact.

## 3 Technological innovation in healthcare: clinical contributions and barriers to real-world translation

Technological innovation has produced significant advances in medical care, giving rise to a new generation of tools that improve diagnostic accuracy and optimize therapeutic interventions, contributing to the advancement of personalized and precision medicine (2, 3). A multitude of emerging and established technologies are currently reshaping the healthcare sector with important implications for patient care, and the long-term sustainability of healthcare systems.

A first cluster of technologies affects how disease is detected, monitored, and managed at the level of the individual patient and the health system. This group includes telemedicine and health information systems, AI applied to diagnostics and clinical decision support, wearable devices and mobile health platforms, Big Data analytics and digital twins, and virtual and augmented reality for training. Their common thread is the generation, integration, and use of clinical data to support earlier, more accurate, and more personalized care. Key examples, applications, and implementation barriers of these technologies are summarised in Table 1.

**Table 1:** Representative examples of emerging healthcare technologies, their principal clinical applications, potential contributions to healthcare delivery and sustainability, and major barriers to large-scale implementation and real-world translation.

Technology	Main clinical applications	Potential contributions	Main barriers to large-scale implementation	Key references
Telemedicine	Remote consultations, chronic disease monitoring, emergency care	Improved healthcare accessibility, continuity of care, reduction of geographical barriers	Uneven infrastructure, reimbursement limitations, variability in quality and access, incomplete long-term integration after COVID-19	(4–6, 28, 29)
Electronic medical records and health information systems	Clinical documentation, medication safety, workflow integration	Improved communication across care pathways, support for clinical decision-making, reduced administrative burden	Fragmented and non-interoperable systems, cybersecurity requirements, implementation complexity	(7)
Artificial intelligence (AI)	Medical imaging, decision support, biomarker discovery, drug development	Earlier diagnosis, support for personalized medicine, optimization of clinical research and healthcare efficiency	Algorithmic bias, limited generalisability, explainability challenges, regulatory uncertainty, clinician adoption barriers	(8–14, 27)
Wearables and mHealth	Continuous monitoring, prevention, chronic disease management	Longitudinal patient data, earlier intervention, patient empowerment	Low long-term adherence, rapid user dropout, partial integration into clinical workflows, data reliability concerns	(15–18, 65, 94–96)
Big Data analytics and digital twins	Predictive modelling, population segmentation, healthcare planning, in silico simulation	Evidence-based decision-making, proactive healthcare management, clinical risk prediction	Data governance challenges, interoperability limitations, computational complexity, ethical concerns	(19–24)
Virtual and augmented reality	Medical training, surgical simulation, rehabilitation	Improved procedural training and patient safety	Infrastructure costs, implementation complexity, uncertain scalability	(25, 26)
Surgical robotics	Minimally invasive surgery, rehabilitation, assisted mobility	Greater precision, improved postoperative recovery, reduced hospitalization	High capital costs, specialised training requirements, variable evidence according to indication	(30–33, 41, 42)
3D printing and bioprinting	Personalized implants, prostheses, surgical planning, regenerative medicine	Patient-specific solutions, anatomical modelling, tissue engineering potential	Pre-clinical stage for many applications, evolving regulatory frameworks	(34–36, 43)
Biomaterials and nanomedicine	Tissue engineering, targeted drug delivery	Precision therapeutics, controlled drug release, reduced systemic adverse effects	Long development timelines, demanding toxicology and regulatory requirements	(37–40, 44, 45)

In this context, telemedicine and digital health platforms have expanded opportunities for remote consultations, continuous patient follow-up, chronic disease management, and proactive health monitoring, particularly highlighted during the COVID-19 pandemic (4–6). By facilitating a more fluid communication between professionals and patients, while reducing geographical barriers, these technologies have contributed to improving healthcare accessibility and continuity of care. The growing digitalisation of healthcare delivery has also accelerated the implementation of interoperable electronic medical records, automated administrative workflows, and integrated clinical systems, aimed at improving communication across care pathways and supporting clinical decision-making (7). Together, these systems facilitate clinical documentation, medication safety, and the integration of increasingly complex healthcare information.

The increasing availability and integration of clinical data has, in turn, facilitated the expansion of AI-driven applications in diagnostics, clinical decision support, biomarker discovery, and drug development (8–14). In particular, AI algorithms applied to medical imaging have demonstrated diagnostic performance comparable to specialist clinicians in selected controlled settings, supporting earlier and more efficient detection of pathological findings (11). Beyond diagnostics, AI-based approaches are increasingly used to analyse genomic and proteomic information, optimize individualized treatment strategies, and accelerate pharmaceutical research and clinical trial design (12–14).

At the same time, wearable devices and mobile health applications are extending this digital ecosystem into people's daily lives. Smartwatches, fitness trackers, and related monitoring technologies enable the continuous collection of physiological and behavioural data, potentially supporting disease prevention, chronic disease management, and earlier clinical intervention (15–18). At a broader system level, the combination of Big Data analytics, predictive modelling, and digital twins is enabling new approaches to population segmentation, proactive healthcare planning, and evidence-based decision-making (19–24). Similarly, virtual and augmented reality are increasingly incorporated in medical training, surgical simulation, medical skills evaluation, and patient rehabilitation (25, 26).

Taken together, the technologies in this first cluster have demonstrated measurable benefits in controlled and pilot settings. However, translating these results into consistent, system-wide impact faces persistent barriers. Common barriers include fragmented and non-interoperable health information systems, difficulties integrating digital tools into routine clinical workflows, uneven long-term patient engagement, and concerns regarding algorithmic bias, explainability, generalisability, and data governance (27). Telemedicine deployment, although accelerated during the COVID-19 pandemic, has also shown substantial variability in quality, accessibility, and long-term consolidation across healthcare systems, including within Europe and Spain (28, 29). The broader structural and organisational factors that condition the large-scale adoption and sustainability of healthcare innovation are discussed in Section 5.

A second cluster of technologies operates at the level of therapeutic intervention, modifying how disease is treated rather than how it is detected or managed at a distance. This group includes surgical robotics, three-dimensional printing, biomaterials, and nanomedicine. Within this group, surgical robotics has demonstrated improved clinical outcomes in selected procedures, with documented benefits in precision, minimally invasive approaches, and postoperative recovery (30–33). In parallel, three-dimensional printing and bioprinting have advanced the manufacture of personalized medical devices, including custom-fit prostheses, implants, and patient-specific anatomical models for surgical planning (34–36). Closely related is the field of biomaterials, which supports increasingly precise and personalized therapeutic strategies, including targeted drug delivery systems and tissue engineering applications (37–40). Additional examples and implementation barriers for these technologies are summarised in Table 1.

Despite their considerable scientific and clinical potential, the large-scale clinical translation of these technologies remains limited by substantial implementation barriers. Surgical robotics systems involve very high capital costs that restrict access to well-resourced centres, while evidence regarding outcome advantages varies substantially according to indication and comparator (41, 42). Similarly, three-dimensional bioprinting remains largely pre-clinical for soft-tissue applications, with regulatory frameworks for bioprinted constructs still under development in Europe and elsewhere (43). Biomaterials and nanomedicine applications also face extended development timelines and demanding regulatory requirements, including specific biocompatibility and toxicology testing that contribute to the translational bottlenecks discussed in subsequent sections (44, 45). Overall, the translational gap between laboratory demonstration and routine clinical implementation remains particularly pronounced for this therapeutic cluster.

The review of these functional clusters reveals a consistent pattern: evidence for clinical benefit in controlled settings is generally positive, but the conditions required for translating that benefit into sustainable, system-wide impact are frequently unmet. Benefits tend to be demonstrated in specialised and well-resourced contexts, whereas large-scale adoption requires additional infrastructure, organisational adaptation, regulatory clarity, and long-term implementation capacity that are often absent. In this context, the realization of the potential of these technologies is not automatic, but depends on strategic planning, robust regulatory frameworks, adequate funding, and the institutional capacity to integrate new tools safely and equitably. These systemic challenges are particularly relevant in the European and Spanish context and are examined in detail in the following sections.

## 4 Medical technology development and barriers to clinical translation

Despite substantial technological advances and promising early-stage results, many healthcare technologies fail to achieve large-scale clinical implementation or sustainable real-world impact. A major determinant of this translational gap is the complexity of the medical technology development and regulatory cycle. In this context, ensuring patient safety remains the bedrock of medical regulation. For medical devices—encompassing software, hardware and integrated systems—this regulatory framework critically aims to confirm their safety, efficacy, and performance (46). Devices are typically classified by risk (e.g., Class I for low-risk, Class II for moderate-risk, and Class III for high-risk), with higher-risk categories naturally demanding more strict testing and specifications. While indispensable, this regulatory process is complex, often entailing lengthy and resource-intensive validation procedures. Though designed to protect patients, it can create significant hurdles, with medical innovations facing substantial delays in reaching the market, or, in some cases, being abandoned altogether due to the sheer weight of these regulatory burdens (46, 47).

**Figure 1:**
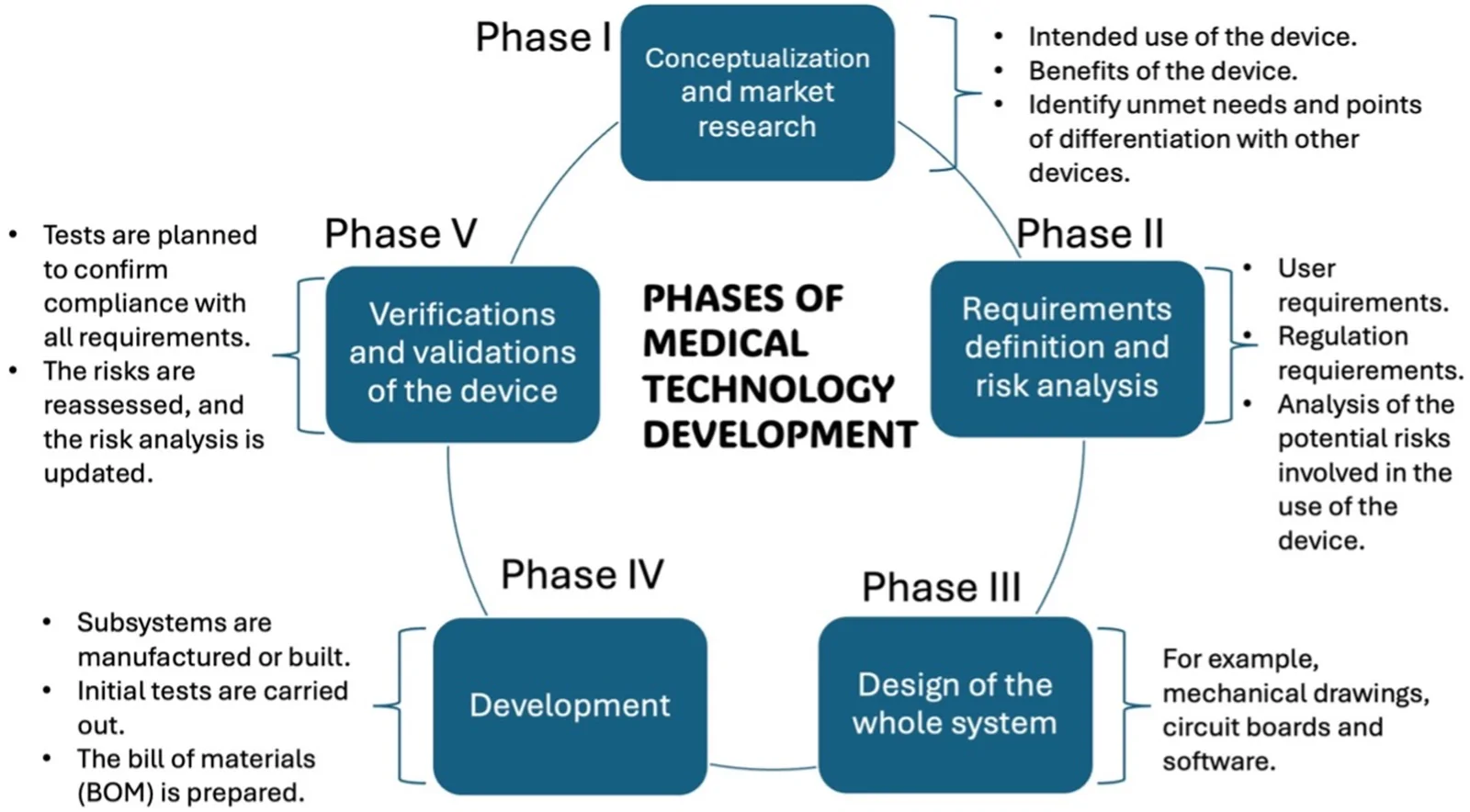
Medical technology development cycle.

This challenging landscape involves a multi-stage development lifecycle (Figure 1), spanning from early conceptualization and risk analysis to iterative prototyping, validation, certification, and eventual clinical deployment. Throughout this process, technologies must progressively demonstrate not only technical feasibility, but also safety, reliability, manufacturability, and clinical utility before large-scale adoption can occur.

Upon achieving a significant development milestone, such as TRL 6, a medical technology confronts an exhaustive battery of tests. These are designed to confirm its safety and compatibility within the demanding medical or hospital environment, including its interaction with both patients and clinical staff. This stage requires a series of stringent validation and verification checks, often including unit tests of individual submodules, which must be performed in certified laboratories to ensure compliance with medical device regulations (48–51). A critical challenge is that many essential safety tests are inherently destructive, meaning prototype units are rendered unusable post-testing. This necessitates the manufacture of multiple units solely for these evaluations, significantly increasing development costs, sometimes compromising the economic viability of particularly expensive or complex technologies. Consequently, many promising technologies fail to progress beyond early validation stages due not only to technical limitations, but also to financial, organisational, or regulatory constraints. To mitigate this substantial impact, considerable upfront investment in material, organisational, and administrative resources is crucial, ideally preceded by the intensive use of virtual predictive models and robust exploitation and business plans to anticipate feasibility and de-risk the endeavour (52).

**Figure 2:**
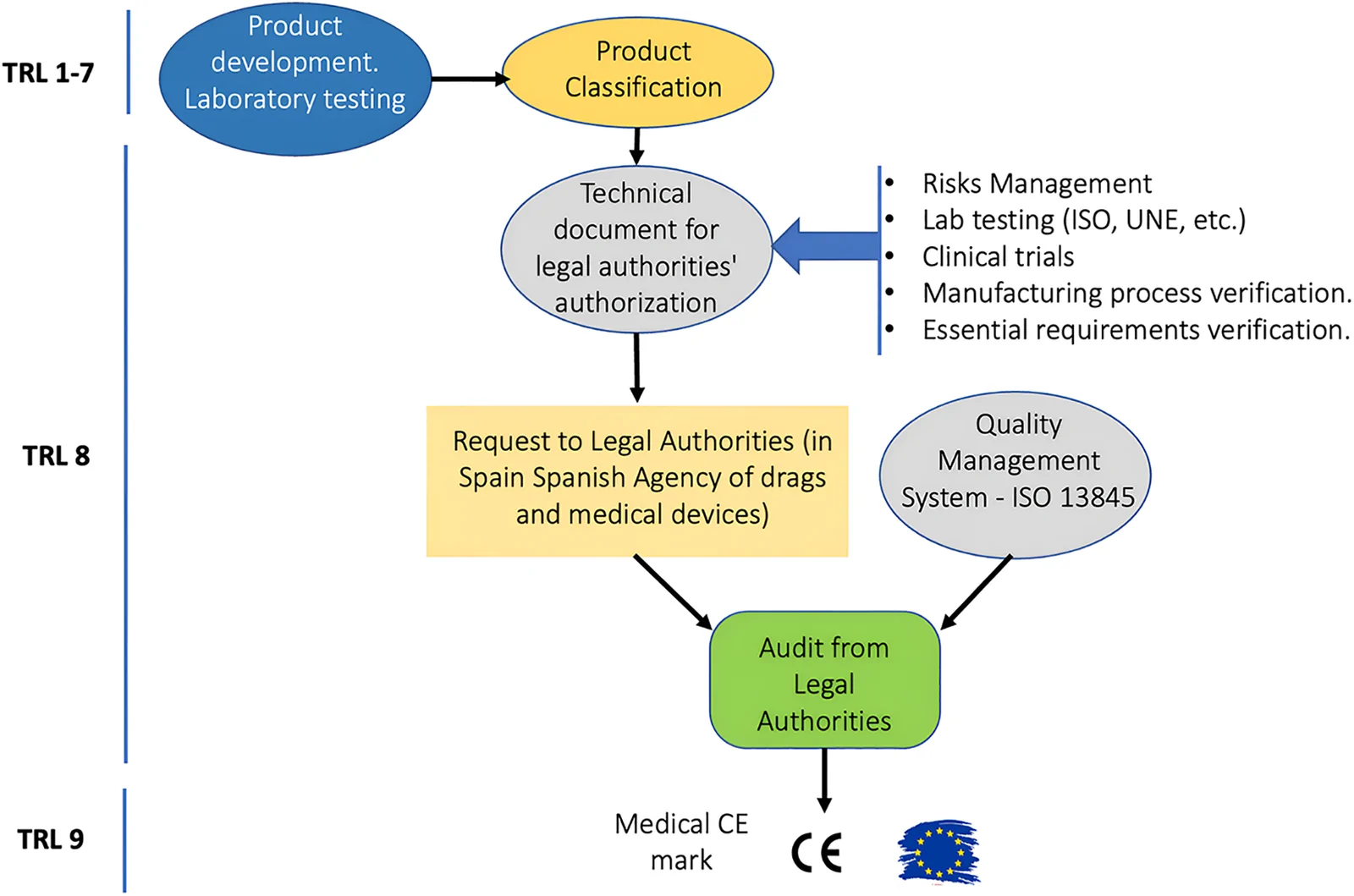
Example of the validation process starting from TRL 7 in Europe-Spain, when the Spanish agency of drugs and medical devices (AEMPS) act as notified body.

The certification process begins once technological development is complete, at approximately TRL 8, where non-healthcare regulations affecting the product must also be met (Figure 2). Once regulatory compliance is achieved, the device typically becomes ready for clinical trials, designed to definitively demonstrate its usability, reliability, efficacy, and, above all, safety in human subjects. These trials must enrol enough participants to ensure statistical significance and account for population variability. If the clinical trial data is positive, a comprehensive dossier detailing the device, including the technical documentation and the clinical evaluation report, is meticulously compiled and submitted to a Notified Body (or equivalent regulatory authority) for exhaustive assessment and, ultimately, certification (53). Functionality, efficacy, manufacturing processes, quality, and traceability are especially affected by current regulations and must be carefully considered in the design and development roadmap to optimize resources, including time as a critical resource. These stages frequently require substantial financial investment, long development timelines, and continuous interaction with regulatory authorities and notified bodies before market authorization can be achieved.

The successful completion of both pre-clinical and clinical validation as well as verification trials and certification provide robust assurance that the medical technology is indeed safe and effective, fulfilling the critical requirements for which it was developed. In this process, Notified Bodies play a pivotal role, acting as crucial gatekeepers to ensure full regulatory compliance of a new technology and the adequacy of its manufacturing processes. Their approval unlocks market entry, allowing the new technology to be deployed.

Medical device regulations vary significantly across regions. In Europe, the Medical Device Regulation (MDR) (54) establishes strict requirements to ensure device safety and efficacy which may increase certification timelines and development costs, particularly for startups and SMEs. This has generated important debate regarding how to balance robust evidence generation and patient safety with the need to maintain innovation capacity and international competitiveness. Additionally, the limited capacity of NBs has created bottlenecks in the certification process and unusual practices (such as the fast track or dedicated assessments in some cases, where the NB prioritizes the application if an extra fee is paid), slowing down innovation (55).

In contrast, the U.S. and China have developed regulatory frameworks with explicit accelerator mechanisms that facilitate earlier market entry, though these approaches involve their own trade-offs between speed and the depth of pre-market evidence requirements. The FDA has continued to modernize its approach to medical device regulation, particularly for software as a medical device (SaMD) (56). The FDA's Digital Health Innovation Action Plan includes initiatives like the Pre-Certification (Pre-Cert) Pilot Program, which evaluates the developer's organisational excellence rather than focusing solely on the product. This approach aims to expedite approvals for innovative digital health products while maintaining safety and efficacy standards. Guidance like AAMI TIR45 outlines how agile practices can comply with regulatory standards such as FDA 21 CFR Part 820 and ISO 13485, enabling faster prototyping, testing, and iteration. The FDA offers different certification pathways depending on the device's risk level, including the 510(k) pathway for low-risk devices and the premarket approval (PMA) for more disruptive innovations.

Similarly, China's National Medical Products Administration (NMPA) has implemented reforms to streamline medical device approvals, particularly for innovative devices. The Special Review Procedure for Innovative Medical Devices and the Priority Approval Procedure allow expedited reviews for high-impact devices in areas like rare diseases, AI diagnostics, and radiation therapy (57). The NMPA's fast-track pathways are selective but have significantly reduced approval times for qualifying devices. For example, the average evaluation time for innovative devices has improved by 83 days. These pathways also align with international standards (e.g., ISO, FDA) while addressing local health needs. Recent regulatory changes under the new Medical Devices Administration Law further emphasize efficiency and innovation by allowing transferability of market authorization certificates and promoting localization through initiatives like Made in China 2025 (58). At the same time, The National Medical Products Administration (NMPA) maintains regulatory requirements that favour domestic manufacturers and often require local clinical evaluation, even for devices already approved in other regions. More broadly, accelerated regulatory pathways continue to generate debate regarding the balance between innovation speed, robustness of clinical evidence, long-term safety monitoring, and healthcare system preparedness for implementation.

The regulatory differences across regions shape the expansion strategies of companies in the healthcare sector and influence the adoption of new technologies globally. While the U.S. and China have developed accelerator mechanisms for market entry, this speed advantage involves trade-offs: post-market surveillance data suggest that faster pre-approval pathways can, in some device categories, result in higher rates of subsequent safety-related actions. Europe, with the MDR framework, faces the distinct challenge of maintaining rigorous safety standards while addressing documented bottlenecks in Notified Body capacity and certification timelines that disproportionately affect smaller innovators (Table 2).

**Table 2:** Comparison of the certification processes in Europe, China, and the U.S.

Aspect	**Europe**	**U.S.**	**China**
Law	Medical Device Regulation (MDR)	Drugs and Cosmetics Act Title, 21-Code of Federal Regulations	State Order No. 739 and other NMPA orders
Classes	I, IIa, IIb, III	I, II, III	I, II, III
Approval time	Lengthy process with recent delays	Long but predictable process	Variable
Innovation support	Moderate (no specific certifications)	High (breakthrough device designation)	Evolving
Local market access	Strict procedural regulation	Competitive	Local protectionism
Regulatory body	Fragmented (Notified Bodies)	Single (FDA)	Single (NMPA)
Language of submission	Covering European Languages	English	Chinese
Quality Management System	ISO 13485	21 CFR 820	ISO 13485 or Chinese GMP
Validity	Depends on declaration of conformity	Unexpired	5 years (for class I unexpired)
Significant product change	Report to notified body with evidence	New 510k	New change registration
Costs	High	Very high	Medium

## 5 From innovation to implementation: healthcare ecosystems, technology transfer, and sustainability

The capacity for innovation in healthcare systems is fundamental not only to their long-term sustainability but also to guarantee their ability to adapt to new challenges, such as population aging, the emergence of new diseases, and the rapid evolution of new technologies and therapies (59, 60). However, the real-world impact of healthcare innovation depends less on technological advances themselves than on the capacity of healthcare ecosystems to support validation, regulation, implementation, technology transfer, workforce adaptation, and large-scale adoption within routine clinical practice (61, 62).

Europe possesses a strong scientific and technological base in healthcare innovation. In 2024, the European medical device sector represented approximately 26% of the global market, with a total value of €160 billion, generated around 16,000 applications submitted to the European Patent Office, and provided 880,000 jobs across the continent (63). Nevertheless, the translation of scientific and technological innovation into scalable healthcare impact remains uneven across Europe, including in countries with strong industrial and research ecosystems. Although countries such as Germany, France, and the United Kingdom concentrate a substantial proportion of industrial capacity, patent activity, and technology transfer infrastructure, healthcare systems across Europe continue to face important challenges related to implementation, interoperability, regulation, reimbursement, and large-scale adoption of emerging technologies (64–66). Within this context, Spain represents a particularly illustrative case. Despite ranking fifth in European medical device market share (6.3%) (63), Spain continues to face important limitations in patent generation, technology transfer capacity, and workforce specialization (67). These differences suggest that the principal challenge for many European healthcare systems is often not the generation of scientific innovation itself, but the ability to translate it into financially viable, durable, and equitably distributed healthcare solutions.

The translational gap between innovation and implementation is perhaps most evident in technology transfer processes. Academic institutions play a central role in the development of emerging healthcare technologies, yet technical feasibility within research environments rarely guarantees successful clinical adoption. Many innovations stagnate during the transition between academic development and healthcare implementation because regulatory pathways, usability constraints, market adoption strategies, and reimbursement conditions are addressed too late in the development process (68). In many cases, insufficient implementation-oriented funding, weak public-private collaboration, and limited institutional support through Technology Transfer Offices (TTOs) prevent promising academic innovations from progressing beyond early development stages. Successful technology transfer therefore requires far more than scientific quality: it depends on sustained funding, industrial collaboration, implementation planning, regulatory expertise, workforce readiness, and institutional structures capable of supporting long-term clinical implementation. This translational gap is particularly relevant for startups and early-stage technologies, which often lack the financial and organisational capacity required to navigate validation, certification, procurement, reimbursement, and integration into healthcare systems.

**Figure 3:**
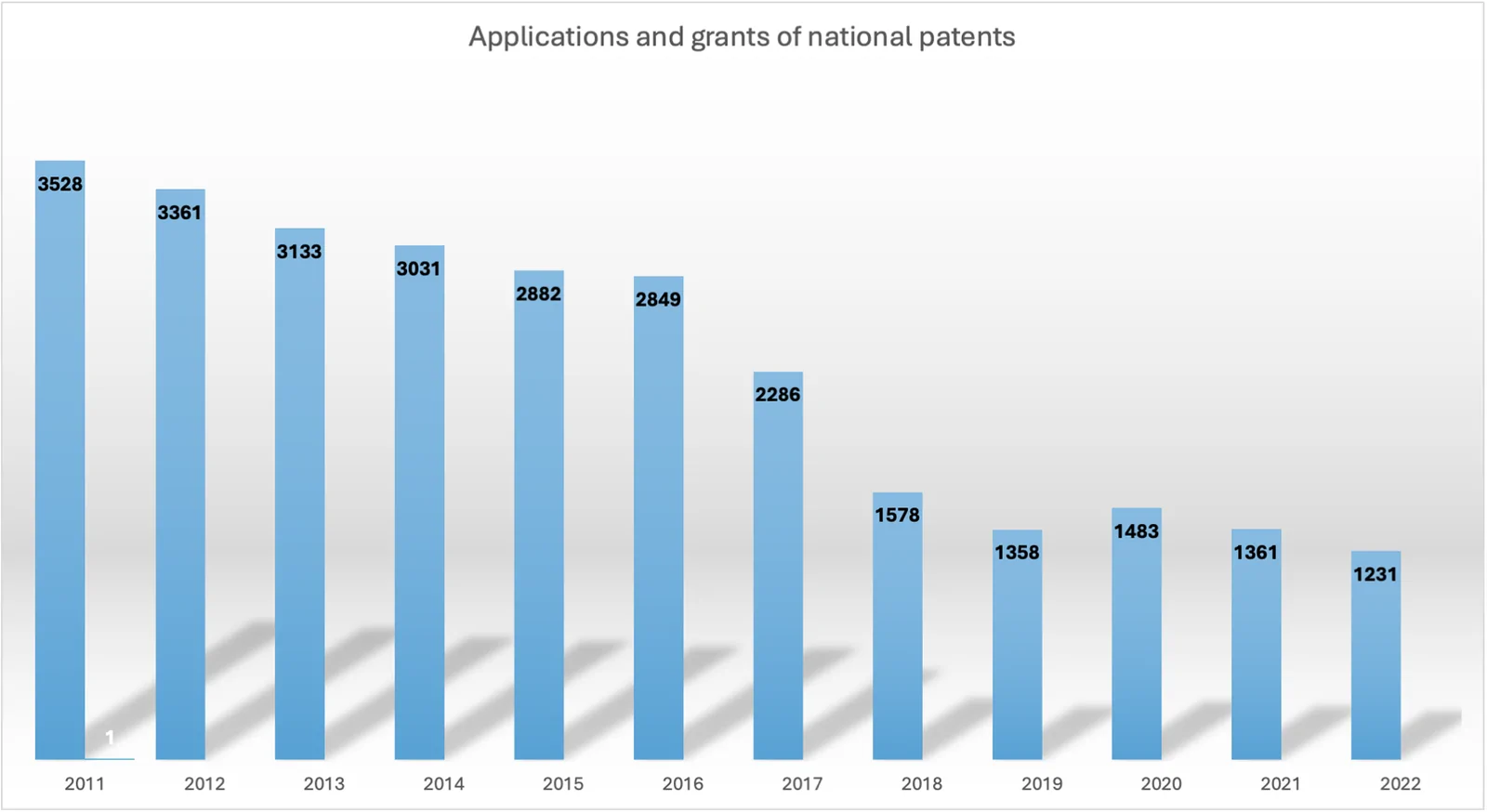
Evolution of national patent applications filed by Spanish public universities as a percentage of the total in Spain (2011–2022). Adapted with permission from “Evolución de las solicitudes de patentes en los últimos 12 años”, by the Spanish Patent and Trademark Office, *La OEPM en cifras (2022).*

**Figure 4:**
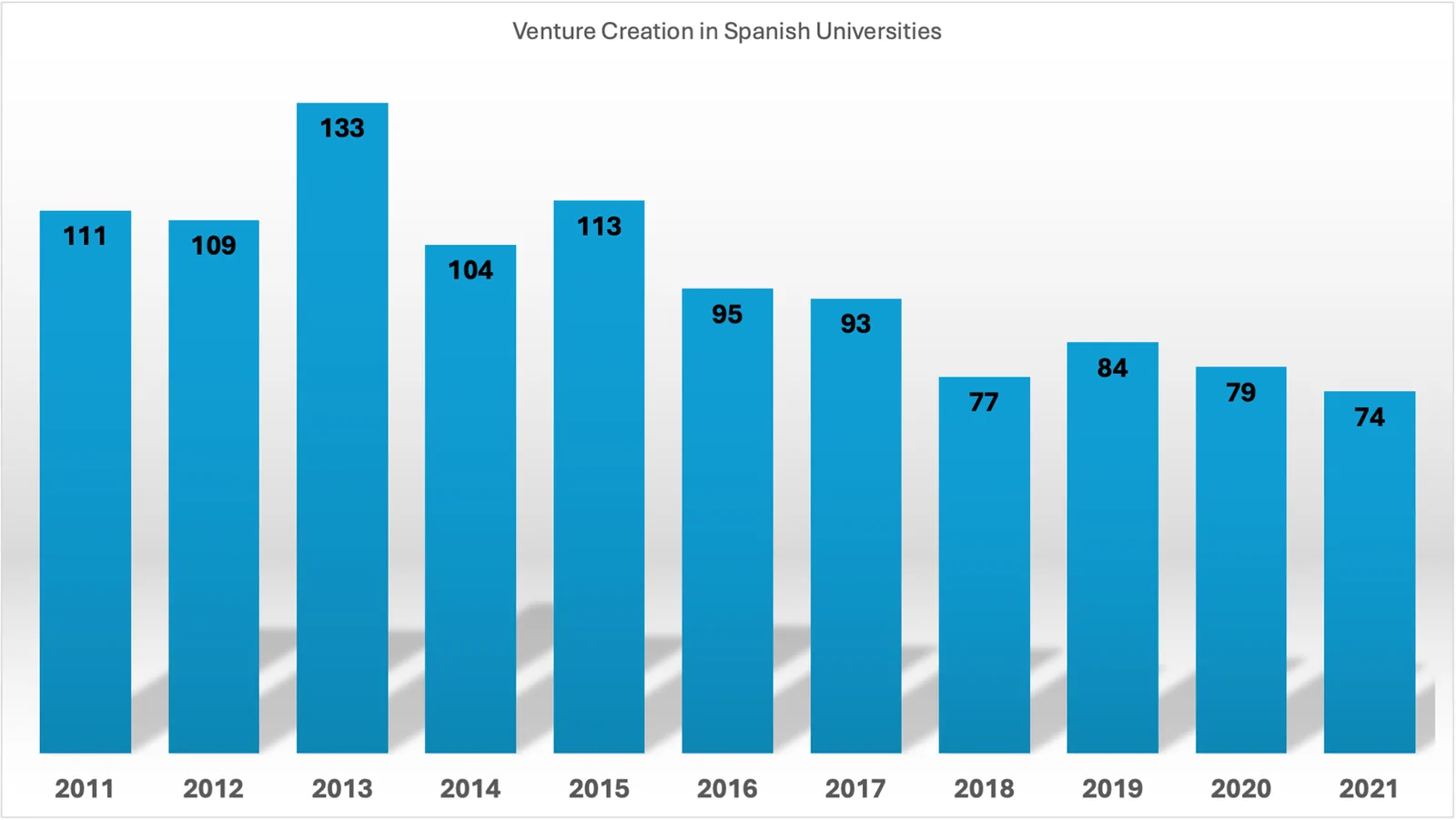
Evolution of spin-off creation in Spanish universities (2011–2021). Adapted with permission from “SPIN-OFF ACADÉMICOS (I)” by the Conference of Rectos of Spanish Universities, *Research, Knowledge Transfer and Scientific Culture in Spanish Universities Report. I+TC+D Survey, RedOTC, R&D&I Sectoral Committee, Crue Spanish Universities.*

**Figure 5:**
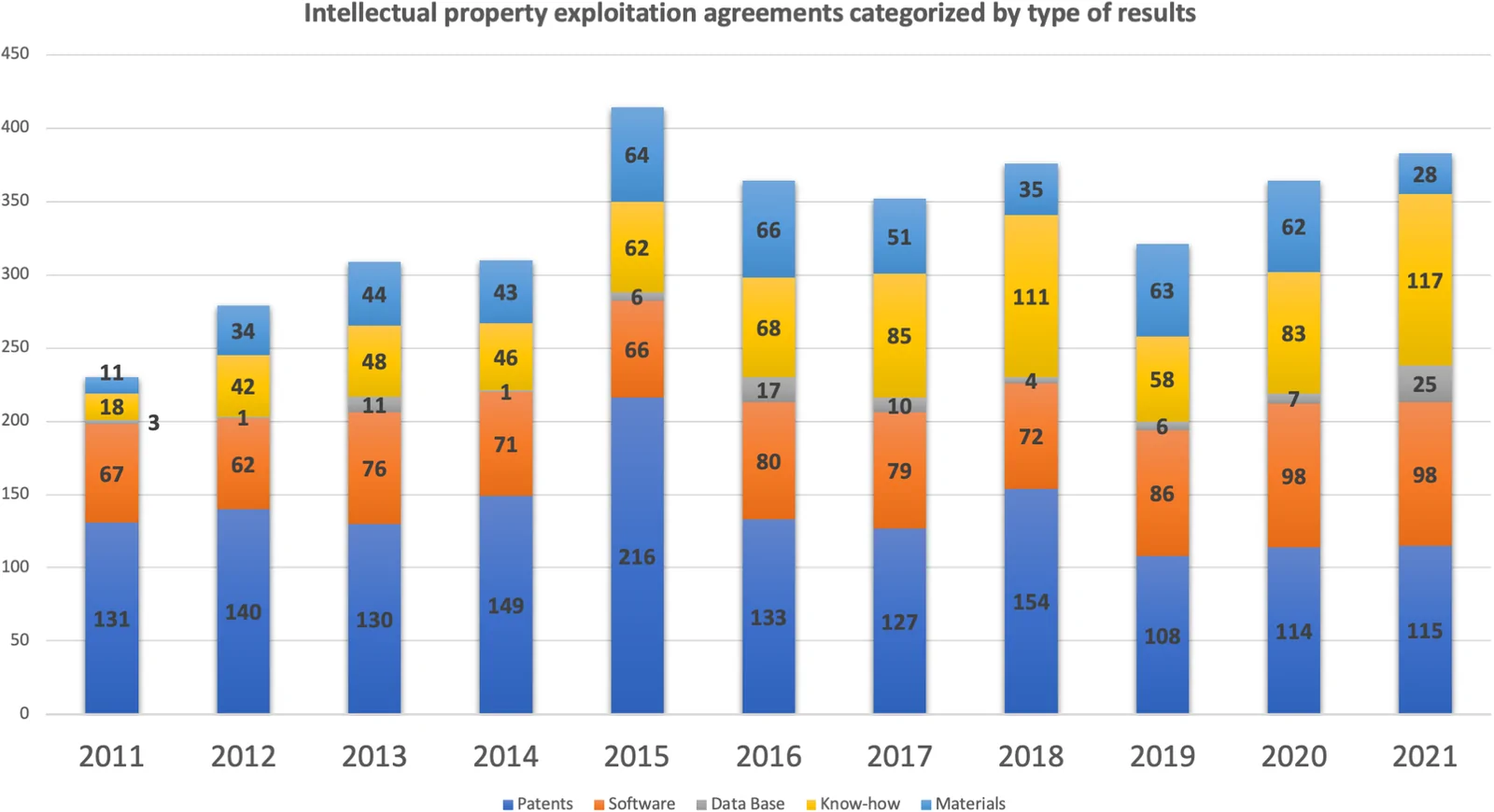
Distribution of the number of licenses by type of innovation (2011–2021). Adapted with permission from “Acuerdos de explotación de PI, según tipo de resultados” by the Conference of Rectos of Spanish Universities, *Research, Knowledge Transfer and Scientific Culture in Spanish Universities Report. I+TC+D Survey, RedOTC, R&D&I Sectoral Committee, Crue Spanish Universities.*

In response to these translational and implementation challenges, Europe and particularly Spain have experienced sustained growth in investment initiatives targeting deep tech, digital health, diagnostics, and AI-driven healthcare technologies, supported by both specialised private investment ecosystems and large European funding programs such as Horizon Europe and the European Innovation Council (EIC). Although these initiatives have strengthened the European innovation landscape and reduced historical dependence on U.S.-based investment ecosystems, important limitations remain in transforming scientific advances into sustainable companies and broadly implemented healthcare solutions. These limitations are reflected in recent innovation trends across Europe and particularly in Spain. Although patent applications in the healthcare sector have increased in recent years (Figure 3), this growth has not been accompanied by a proportional increase in spin-off creation (Figure 4) or technology licensing activity (Figure 5). This divergence reflects persistent difficulties in converting scientific advances into scalable healthcare solutions and sustainable industrial development. One important bottleneck emerges during intermediate translational stages, where investment needs exceed traditional academic or seed funding despite persistent technological and regulatory uncertainty. This financing gap, commonly referred as the “valley of death,” continues to represent one of the principal causes of failure among healthcare spin-offs and early-stage deep tech companies (69). In Spain, this translational bottleneck is particularly pronounced. Technology transfer structures and innovation policies remain insufficiently aligned with implementation needs, and researchers often face limited institutional support, implementation resources, and specialised commercialisation expertise when attempting to translate innovations into practice (70). In response, the European Union has implemented initiatives such as Horizon Europe, Research and Innovation Actions (RIA), and the EIC Scale-Up program, which increasingly aim to strengthen translational development, facilitate industrial participation, and support implementation-oriented innovation ecosystems (71). Similarly, public-private partnerships have emerged as important mechanisms for accelerating validation processes and facilitating access to technological expertise, industrial infrastructures, and implementation resources (72). Nevertheless, important limitations persist in the coordination between scientific innovation, healthcare implementation, regulatory adaptation, procurement systems, and market integration, particularly for small and medium-sized enterprises and early-stage healthcare technologies. Strengthening TTOs, implementation-oriented funding mechanisms, workforce training, and public-private collaboration therefore becomes not only an economic objective, but a necessary condition for sustainable healthcare innovation.

Beyond these structural and translational barriers, healthcare innovation also raises important challenges related to equity and accessibility. Although digital health technologies may improve chronic disease management, preventive care, and patient participation (73),, access to these benefits remains highly dependent on digital literacy, infrastructure availability, technological access, and interface accessibility (74). Consequently, innovation strategies that fail to incorporate equity considerations from the outset may inadvertently concentrate the benefits of healthcare innovation in well-resourced institutions while leaving vulnerable groups behind. In this sense, equity should not be treated as a downstream consequence of implementation, but as a core dimension of sustainable healthcare innovation.

Eventually, the sustainability of healthcare innovation depends less on the existence of promising technologies than on the capacity of healthcare systems to absorb them. The European and particularly the Spanish experience illustrates that scientific output, technological sophistication, and regulatory approval alone are insufficient to guarantee healthcare impact. Sustainable innovation therefore requires alignment between regulation, infrastructure, reimbursement, workforce preparedness, and technology transfer in order to ensure that the benefits of innovation reach the broader population rather than remaining concentrated in well-resourced settings.

## 6 Data governance and trust in AI-driven healthcare ecosystems

The large-scale implementation of AI-driven healthcare depends not only on technological performance, but also on the existence of governance frameworks capable of supporting trustworthy, interoperable, secure, and socially acceptable uses of health data. The accelerating digitalisation of healthcare systems, together with the widespread adoption of telemedicine platforms, wearable devices, remote monitoring systems, and other digital health technologies, has substantially expanded the generation and circulation of health-related data across healthcare ecosystems (75). These data ecosystems create opportunities for more personalized, preventive, and continuous healthcare delivery, while also supporting remote monitoring, clinical decision-making, and the generation of real-world clinical evidence. However, the capacity of healthcare systems to translate these technological capabilities into sustainable healthcare impact remains highly dependent on how data governance, interoperability, privacy, accountability, and patient trust are operationalized within real-world clinical infrastructures.

One of the principal challenges concerns the fragmentation of health data across institutions, platforms, and regulatory environments. Clinical information frequently remains distributed among hospitals, research centres, private companies, and regional healthcare systems using heterogeneous infrastructures and non-standardised formats. Accordingly, limited interoperability not only restricts continuity of care and secondary data use, but also hinders the development, validation, and implementation of AI-based healthcare solutions (76). Consequently, the value of health data depends not simply on data availability, but on the existence of governance models capable of enabling secure, traceable, and ethically acceptable data integration across institutions and jurisdictions. Large-scale initiatives such as UK Biobank and the U.S. The Cancer Imaging Archive (TCIA) illustrate the potential of shared and structured data infrastructures for supporting biomedical research, AI development, and secondary use of clinical information under regulated governance frameworks (77). Similarly, initiatives such as the European Health Data Space (EHDS) seek to facilitate more coordinated and interoperable data-sharing frameworks while preserving privacy and regulatory oversight (78). Nevertheless, important uncertainties remain regarding the practical implementation, standardization, governance complexity, and long-term sustainability of these large-scale infrastructures.

These tensions become particularly evident in debates surrounding data sovereignty, consent, and patient control over health information. Although patients are increasingly recognized as the legitimate owners of their health data, operationalising meaningful control over data access and secondary use remains difficult within fragmented healthcare ecosystems (79, 80). Dynamic consent platforms, federated data models, and privacy-preserving analytical approaches such as homomorphic encryption and advanced anonymization techniques have been proposed as potential mechanisms to facilitate secure data sharing without requiring direct data centralization (81). These approaches aim to improve transparency, traceability, and patient control over secondary data use while helping preserve privacy across distributed healthcare infrastructures. Similarly, concepts such as “data donation” have emerged as possible frameworks for supporting scientific research through voluntary citizen participation under transparent and ethically regulated condition (82). However, these approaches also introduce important unresolved questions regarding governance complexity, consent fatigue, accountability, commercialisation of health data, and the unequal capacity of institutions to maintain secure and transparent data infrastructures. Similar debates have emerged around blockchain-based models for health data sharing, which have been proposed as potential mechanisms for improving transparency and traceability but also illustrate the unresolved tensions surrounding interoperability, governance complexity, scalability, consent management, and public trust in digital health infrastructures (83). These models additionally require robust mechanisms for transparency, independent oversight, and periodic auditing in order to prevent misuse, unauthorised commercialisation, or inequitable exploitation of health data. In this context, trust becomes not only an ethical issue, but a prerequisite for sustainable implementation of AI-driven healthcare systems.

The increasing use of real-world data (RWD) further reinforces these governance challenges. Compared with randomised controlled trials, real-world clinical data may provide broader and more representative information regarding treatment effectiveness, long-term outcomes, healthcare utilization, and patient heterogeneity. Consequently, RWD is increasingly used to support clinical research, pharmacovigilance, healthcare planning, and AI model development. However, expanding secondary uses of health data also intensifies tensions between scientific innovation, public benefit, commercial interests, and privacy protection. Although health data are increasingly framed as a strategic public resource, healthcare systems continue to face major difficulties in balancing data accessibility with transparency, accountability, and citizen trust. These challenges are particularly relevant in AI-driven healthcare, where model performance frequently depends on access to large, heterogeneous, and continuously updated datasets.

Within this context, patient empowerment—a paradigm where individuals play an active and informed role in decisions concerning their health— should not be understood exclusively as a technological outcome, but rather as a conditional and uneven process shaped by healthcare infrastructures, digital literacy, institutional trust, and accessibility. Although the potential benefits of digital health technologies for patient participation, chronic disease management, and preventive care have been discussed previously, the effective exercise of this autonomy depends heavily on patients’ ability to access, understand, and manage increasingly complex digital information. Consequently, the benefits of data-driven healthcare may remain unevenly distributed across populations and healthcare systems, potentially reinforcing existing social, economic, geographic, and digital inequalities. These asymmetries are particularly relevant for vulnerable populations, whose participation in digital healthcare ecosystems may be limited by barriers related to connectivity, accessibility, health literacy, disability, or institutional support. As a result, patient agency becomes not only a question of technological availability, but also of governance capacity and equitable healthcare implementation.

The growing autonomy of AI systems in healthcare additionally introduces important questions regarding explainability, accountability, and clinical responsibility. AI-driven systems are increasingly involved in diagnostic support, treatment recommendations, risk prediction, and automated clinical workflows, yet many of these models remain difficult to interpret for both clinicians and patients. Under these conditions, transparency and explainability become essential not only for regulatory approval, but also for maintaining professional accountability and patient trust (84). At the same time, explainability alone may not fully resolve broader concerns related to algorithmic bias, generalisability, data quality, or overreliance on automated decision-making systems. Consequently, ethical implementation of AI-driven healthcare requires governance approaches capable of balancing technological innovation with clinical oversight, professional responsibility, patient autonomy, and long-term social trust.

Ultimately, the sustainability of AI-driven healthcare depends less on data generation itself than on the capacity of healthcare ecosystems to govern data use in ways that are interoperable, transparent, secure, equitable, and socially legitimate. Without governance models capable of balancing innovation, privacy, accountability, interoperability, and patient trust, even technically robust AI systems may struggle to achieve sustainable integration into routine healthcare practice.

## 7 Clinical responsibility and accountability in AI-driven healthcare

As AI systems become increasingly integrated into clinical workflows, healthcare decision-making is progressively evolving from a model centred exclusively on human judgment toward more distributed and technologically mediated forms of clinical responsibility. AI-driven systems are already involved in diagnostic support, risk prediction, workflow prioritisation, treatment selection, and remote patient monitoring across multiple healthcare settings. This evolution increasingly complicates the distribution of responsibility, accountability, clinical oversight, and patient safety between healthcare professionals, institutions, manufacturers, and algorithmic systems.

One of the principal difficulties concerns informed consent and patient autonomy in AI-mediated healthcare environments. Although patients are increasingly exposed to algorithm-assisted decision-making processes, the operational logic underlying many AI systems remains difficult to interpret for both clinicians and patients. Consequently, patients may face important limitations in understanding how diagnostic, prognostic, or therapeutic recommendations are generated, particularly when highly complex or continuously adaptive systems are involved (85, 86). These tensions become even more pronounced in technologies involving continuous interaction between algorithms and patients, such as brain computer interfaces (BCIs), adaptive therapeutic systems, or autonomous monitoring platforms (87, 88), where clinical decisions may evolve dynamically in response to continuously generated physiological data. Maintaining meaningful informed consent therefore becomes increasingly difficult, particularly when AI systems evolve dynamically over time or operate with limited explainability.

The growing influence of AI systems on clinical decision-making additionally raises important questions regarding the distribution of authority between algorithms, healthcare professionals, and patients. Although AI systems are generally positioned as support tools rather than replacements for clinical judgment, the integration of algorithmic recommendations into routine workflows may progressively influence diagnostic reasoning, therapeutic choices, and clinical prioritisation processes. Consequently, tensions may emerge between algorithmic outputs and physician autonomy, particularly in contexts characterized by increasing workload pressures, limited time for independent verification, or institutional incentives favouring automation. In practice, excessive dependence on AI-generated recommendations may introduce new forms of automation bias and reduce critical clinical oversight. These risks become particularly relevant because many AI systems continue to function as highly opaque or “black-box” models, making it difficult for clinicians and patients to understand the rationale underlying algorithmic conclusions (84). Such opacity may not only reduce trust in AI-assisted decision-making but also hinder widespread clinical adoption and weaken professional accountability. In this context, explainability becomes important not only for regulatory compliance, but also for preserving professional accountability and maintaining trust in clinical decision-making processes.

Beyond interpretability challenges, the safe implementation of AI-driven healthcare additionally depends on the robustness, generalisability, and security of these systems across real-world clinical environments. AI models trained on insufficiently representative datasets may perform unevenly across populations, potentially reinforcing existing disparities in healthcare delivery or generating clinically inappropriate recommendations for underrepresented groups. Furthermore, increasing interconnectivity between medical devices, digital infrastructures, and healthcare networks has expanded cybersecurity risks within healthcare systems. In 2023, 92% of medical institutions in the U.S. reported experiencing some form of cyberattack (89), illustrating the growing vulnerability of digitally connected healthcare environments. As a result, cybersecurity can no longer be considered exclusively a technical issue, but rather a core component of patient safety, healthcare governance, and long-term system resilience. Consequently, AI-driven healthcare systems require not only clinical validation and regulatory approval, but also continuous monitoring, post-market surveillance, infrastructure protection, and mechanisms capable of identifying failures, biases, or security vulnerabilities after deployment (90).

These challenges ultimately converge around the problem of responsibility distribution in AI-mediated healthcare. Current regulatory and legal frameworks continue to place primary responsibility for diagnosis and treatment decisions on healthcare professionals, even when AI systems substantially influence clinical workflows or recommendations. Consequently, physicians remain responsible for verifying algorithmic outputs, interpreting results appropriately, and ensuring that AI tools are used within validated clinical contexts (91, 92). Inadequate professional training, excessive dependence on automated systems, or off-label use of AI technologies may significantly increase clinical liability. At the same time, manufacturers also bear substantial responsibility for the design, validation, safety, explainability, and post-market monitoring of AI systems, including the obligation to clearly define operational limitations and intended use conditions. Healthcare institutions themselves additionally play a critical role by determining procurement practices, implementation protocols, cybersecurity infrastructures, professional training requirements, and oversight mechanisms governing AI deployment within clinical environments.

At the regulatory level, agencies such as the FDA and EMA are increasingly attempting to adapt existing frameworks to the growing complexity and dynamic nature of AI-driven healthcare technologies (93). However, important difficulties remain in balancing innovation, patient safety, clinical evidence requirements, and the rapid pace of technological evolution. These tensions are particularly relevant for continuously learning systems, adaptive algorithms, and highly personalized AI-driven medical devices, where long-term performance may evolve after deployment and where traditional validation models may become insufficient. Yet regulatory approval alone cannot fully address the long-term governance challenges posed by continuously evolving AI systems. Instead, healthcare systems increasingly require continuous governance models capable of integrating clinical oversight, technical monitoring, cybersecurity protection, professional accountability, and post-deployment evaluation throughout the entire lifecycle of AI technologies.

Overall, the implementation of AI-driven healthcare requires clear responsibility structures capable of preserving meaningful clinical oversight, maintaining patient trust, and ensuring that increasingly autonomous technologies remain aligned with professional, ethical, and institutional accountability frameworks. Without these conditions, the expanding role of AI in clinical practice risks generating persistent uncertainty regarding professional accountability, patient protection, and legal responsibility despite continued technological advances.

## 8 Conclusion

The growing incorporation of emerging technologies into healthcare systems is progressively redefining both clinical practice and the organization of medical care. Beyond their technical capabilities, technologies such as AI, digital health platforms, remote monitoring systems, advanced medical devices, and data-driven clinical infrastructures are reshaping how healthcare systems approach prevention, diagnosis, treatment personalisation, patient participation, and long-term system sustainability. Many of these developments are closely aligned with the principles of 4P Medicine by enabling more predictive, preventive, personalized, and participatory models of care. Nevertheless, this review highlights that technological progress alone is insufficient to guarantee meaningful healthcare impact. The successful implementation of healthcare innovation depends fundamentally on the capacity of healthcare systems to support validation, regulation, interoperability, workforce adaptation, technology transfer, governance, and long-term integration into routine clinical practice.

Many of the principal barriers limiting large-scale adoption are therefore not primarily technical, but organisational, regulatory, financial, and institutional. Technologies that demonstrate promising performance under controlled or pilot conditions frequently encounter substantial difficulties when translated into heterogeneous real-world healthcare environments. Fragmented infrastructures, limited interoperability, insufficient implementation-oriented funding, workforce training gaps, cybersecurity vulnerabilities, unclear responsibility distribution, and regulatory complexity continue to constrain the sustainable integration of innovation into routine clinical practice. These challenges are particularly relevant in Europe and Spain, where strong scientific and technological capabilities frequently coexist with more limited translation capacity and persistent barriers to large-scale implementation.

Ultimately, the successful integration of healthcare innovation should not be understood as a purely technological challenge, but rather as a systemic transformation process requiring coordinated governance, institutional readiness, clinical oversight, and long-term implementation capacity. Rather than assuming that technological innovation will automatically generate healthcare transformation, future efforts should focus more critically on understanding the conditions under which technologies can achieve safe, scalable, equitable, and sustainable integration into real-world healthcare systems. From this perspective, the central challenge for contemporary healthcare systems is no longer simply how to develop new technologies, but how to build the organisational, regulatory, and institutional capacity required to integrate them responsibly and effectively into.
