# Correction: Technological innovation in healthcare: challenges, opportunities and impact on sustainability

**DOI:** 10.3389/fdgth.2026.1936077

**Published:** 2026-09-09

**Authors:** María Vallet-Regí, Manuel Doblaré, José A. Garrido, Almudena Fuster-Matanzo, Ángel Alberich-Bayarri, María Elena Hernando, Cecilia E. García Cena, Marie Destarac Eguizabal, Emilio Bouza

**Affiliations:** 1 Universidad Complutense de Madrid, Madrid, Spain; 2 Instituto de Investigación Sanitaria Hospital 12 de Octubre, Madrid, Spain; 3 Fundación Ramón Areces, Madrid, Spain; 4 Centro de Investigación Biomédica en Red en Bioingeniería, Biomateriales y Nanomedicina (CIBER-BBN), Instituto de Salud Carlos III, Madrid, Spain; 5 Instituto Universitario de Investigación en Ingeniería de Aragón (I3A), Universidad de Zaragoza, Zaragoza, Spain; 6 Instituto de Investigación Sanitaria Aragón (IISA), Zaragoza, Spain; 7 ICREA, Barcelona, Spain; 8 Catalan Institute of Nanoscience and Nanotechnology (ICN2), CSIC and The Barcelona Institute of Science and Technology, Barcelona, Spain; 9 Quibim S.L, Valencia, Spain; 10 Centro de Tecnología Biomédica, Universidad Politécnica de Madrid, Madrid, Spain; 11 Universidad Politécnica de Madrid, Escuela Técnica Superior de Ingeniería y Diseño Industrial, Madrid, Spain; 12 Centro de Automática y Robótica (ETSIDI-UPM-CSIC), Madrid, Spain; 13 OWSD Guatemala, Guatemala, Guatemala; 14 ABB Spain, Madrid, Spain

**Keywords:** healthcare, innovation, policies, sustainability, technology

The reference for 97 does not exist and has been removed.

The original version of this article has been updated.

